# Impact of Renin–Angiotensin–Aldosterone System (RAAS) Gene Polymorphism in Essential Hypertension and Antihypertensive Drug Therapy: A Review

**DOI:** 10.1155/ijhy/5530265

**Published:** 2025-05-01

**Authors:** Archana Adhikari, Chandrakala Sharma, Mingma Lhamu Sherpa, Gauthaman Karaunakran, Mona Dhakal, Anita Sharma

**Affiliations:** ^1^Department of Pharmacology, Sikkim Manipal Institute of Medical Sciences (SMIMS), Sikkim Manipal University (SMU), New College Building, 5^th^ Mile Tadong, Gangtok 737102, Sikkim, India; ^2^Department of Biochemistry, Sikkim Manipal Institute of Medical Sciences (SMIMS), Sikkim Manipal University (SMU), Central Referral Hospital Building, 5th Mile Tadong, Gangtok 737102, Sikkim, India; ^3^Government Pharmacy College, Sikkim University (SU), Rumtek, Gangtok 737135, Sikkim, India; ^4^Department of Medicine, Sikkim Manipal Institute of Medical Sciences (SMIMS), Sikkim Manipal University (SMU), Central Referral Hospital Building, 5^th^ Mile Tadong, Gangtok 737102, Sikkim, India; ^5^Indian Institute for Human Settlements, 142, Doon Vihar, Jakhan, Dehradun 248001, Uttarakhand, India

**Keywords:** ACE, AGT, AT1R genes, essential hypertension, gene polymorphism, renin–angiotensin–aldosterone system

## Abstract

Genetic, demographic and environmental factors all play a role in the frequency of an intricate multifactorial condition known as hypertension. Approximately 30% and 50% of BP fluctuation are influenced by genetic variability. Many genetic studies have confirmed the link between genetic variability and susceptibility to essential hypertension; hence, identifying genes associated with essential hypertension susceptibility will aid in understanding the pathophysiology and their influence on how an individual responds towards the antihypertensive therapy. There are also controversial results highlighted in some reports. This review summarises genetic variants of the renin–angiotensin–aldosterone system (RAAS), angiotensinogen (AGT) (M235T), angiotensin converting enzyme (ACE) (insertion/deletion), angiotensin II type 1 receptor (AT1R) (A1166C) and aldosterone synthase (C344T) that are known and might contribute towards the pathophysiology of essential hypertension. Furthermore, the review highlights the response of certain RAAS gene polymorphisms (renin, ACE and AT1R genes) to antihypertensive drugs.

## 1. Introduction

With increased cardiovascular mortality and morbidity, hypertension is a serious concern that has been identified as the primary cause of death in cardiovascular disease across the globe [1–3]. It is a chronic, common and age-related disorder [3–5]. According to some guidelines, hypertension should be identified when a patient's systolic blood pressure (BP) is higher than 140 mm Hg or their diastolic BP (DBP) is higher than 90 mm Hg after routine examination [6]. Disease involving renal and adrenal glands forms the aetiology for the development of secondary hypertension. On the other hand, most cases of hypertension are idiopathic in nature and are known as primary or essential hypertension (EH).

EH reports for around 95% of all cases of hypertension and arises from the intricate interplay between genetic and environmental factors. Genetically, EH contributes to approximately 30% of the variability in BP among individuals [7–10]. The regulation of BP involves a complex system of physiological mechanisms that encompass the balance of extracellular fluid volume, cardiac contractility and vascular tone. These processes are orchestrated by renal, neurological and endocrine systems [10, 11].

EH is influenced by a range of genetic factors, and the renin–angiotensin–aldosterone system (RAAS) plays a crucial role in both its development and management [8, 12, 13]. RAAS is a complex network of hormones that connect the adrenal, renal and cardiovascular systems, regulating fluid electrolyte balance and subsequently atrial BP [14]. Numerous studies have investigated various candidate genes within RAAS. Among approximately 150 genes involved in regulating BP, specific genetic variations within RAAS components such as renin, angiotensinogen (AGT), angiotensin converting enzyme (ACE), angiotensin II type 1 receptor (AT1R) and aldosterone synthase have been directly linked to EH [9, 10, 15, 16].

For instance, renin, released by kidneys, is an essential component of RAAS, initiating the conversion of AGT to angiotensin I (Ang I) [17]. ACE then converts Ang I into the active peptide angiotensin II (Ang II), crucial for maintaining BP and fluid electrolyte balance [15].

The ACE gene exhibits a polymorphism characterised by an insertion/deletion (I/D) of 287 bp ALUya5 element in the intron [2, 15, 18]. Similarly, A1166C polymorphism of the AT1R gene, as well as M235T of the AGT gene and the C344T variant in the aldosterone synthase gene, has been linked to EH in various studies, although results have been conflicting in some cases [15, 16, 19–22].

Furthermore, genetic variations have also been demonstrated to impact how an individual responds to antihypertensive medications [23]. Despite the availability of several antihypertensive drugs, BP control is only accomplished in approximately 50% of treated hypertensive patients worldwide [24, 25]. Genetic factors contribute to roughly half of the variability in response to antihypertensive drugs [5, 26, 27]. BP management strategies thus need to consider an individual's specific genetic makeup [28].

Recent research indicates that genetic variability in the renin gene can influence its activity and production, potentially influencing the response to antihypertensive drugs [17]. ACE inhibitors and AT1R blockers have become increasingly used over the past 2 decades [29]. ACE inhibitors target a zinc metalloproteinase that converts Ang I to Ang II, which results in reducing peripheral vascular resistance, decreased aldosterone secretion and increased natriuresis. They also reduce the breakdown of bradykinin, a vasodilator [2, 6].

In contrast, AT1R blockers block the action of Ang II at the Ang I receptor, leading to vasodilation, decreasing aldosterone production and ultimately reducing BP [30]. Studies have reported notable genetic variation in response to ACE inhibitors and AT1R blockers [31]. For example, the ACE gene polymorphism (I/D) in intron 16 can predict about half of the variability in individual response towards ACE inhibitors, though results may vary across different populations [2, 25, 28, 32]. Similarly, data indicate that A1166C polymorphism of the AT1R gene may be linked with a decreased efficacy of AT1R blockers, although the findings are conflicting among different studies [33–35].

The main objective of this paper is to provide a comprehensive review of association between key genetic variations in the RAAS, such as AGT (M235T), ACE (I/D), AT1R (A1166C) and aldosterone synthase (C344T), and their relationship with EH. Additionally, the paper also examines how antihypertensive medications target RAAS response to renin, ACE (I/D) and AT1R (A1166C) gene polymorphism across various populations.

### 1.1. RAAS Gene Polymorphism and EH

**Figure 1:**
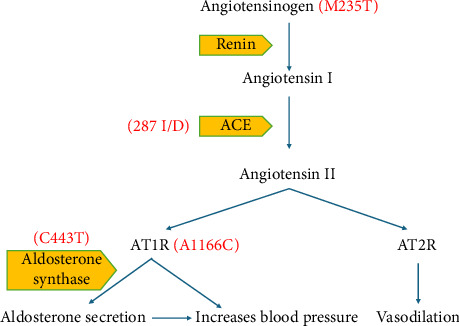
RAAS gene polymorphisms. Explanation for the figure: angiotensinogen (AGT) is cleaved by renin to angiotensin I (Ang I), which is further hydrolysed to angiotensin II (Ang II) by angiotensin converting enzyme (ACE). Ang II has two types of receptors: angiotensin II type 1 receptor (AT1R) and angiotensin II type 2 receptor (AT2R). On one hand, Ang II then binds with AT1R, which directly acts on blood vessels and adrenal glands to increase blood pressure. During this process, aldosterone synthase helps in the secretion of aldosterone that facilitates the reabsorption of sodium and water, thereby increasing BP. On the other hand, AT2R is responsible for vasodilation. There are many gene polymorphisms seen in the RAAS system, such as AGT (M235T), ACE (insertion/deletion) (I/D), Ang II Type 1 receptor (AT1R) (A1166C) and aldosterone synthase (C344T).

As potential genes for EH, RAAS components have been thoroughly investigated. Some studies have shown a significant link between gene polymorphisms of AGT (M235T), ACE (I/D), AT1R (A1166C) and aldosterone synthase (C344T) (Figure 1) with EH [16, 20–22, 36].

#### 1.1.1. AGT

The enzymatic route of the RAAS uses a particular precursor called AGT to produce all angiotensin peptide derivatives [37]. The human AGT gene, which is part of the serpin gene superfamily and has four introns and five exons totalling 13 kb, is located at lq42-43. The renin–AGT enzymatic process, which is a crucial process in the RAAS for controlling BP, uses AGT as a substrate [25].

##### 1.1.1.1. M235T Gene Polymorphism and EH

The AGT region harbours multiple polymorphisms, with particular attention given to the M235T located in exon 2 [25]. The link between the M235T AGT TT gene polymorphism and EH was initially observed in the early 1990s [38]. Since then, numerous studies worldwide have explored this association.

A large-scale study conducted from 2008 to 2013 in 7697 individuals from Yogyakarta Province, Indonesia, showed a positive relationship between M235T and EH [39]. Another study involving 354 participants from the Electricity Generating Authority of Thailand found a similar link between a specific genetic variation (M235T polymorphism) and an increase in BP. This finding emerged from a cohort study with a 5-year follow-up with data collected at two time points—baseline in 2013 and follow-up in 2018. In both of these studies, individuals carrying the T allele among this population were at a higher risk of developing hypertension [40]. Similar were the results in a study conducted among the Mexican population [41].

While all of these studies involved a considerable sample size, however, large-scale studies may yield a better understanding of genetic variation and its impact on EH. A meta-analysis of larger-scale studies conducted among populations worldwide also demonstrated that individuals with the T allele have a higher propensity for EH [42].

While numerous studies across diverse populations have established a positive association between the M235T polymorphism and EH, some studies have yielded conflicting results, indicating no significant relationship between the two (Table 1). For instance, a case–control study involving 406 individuals aged 20–79 years revealed polymorphism in both cases and controls, indicating no significant association with EH [48]. Some of the older research conducted before 2015 in some populations of Mongolia, Calabar, Uyo and Germany also showed negative or no relationships between polymorphism and EH [22, 39, 49].

#### 1.1.2. ACE

ACE is a crucial enzyme in RAAS; it converts Ang I to Ang II, which is a potent vasoconstrictor and is present in blood and many bodily fluids [50, 51]. It is encoded by the ACE gene, located in chromosome 17 (17q23.3), which spans 21 kb and consists of 25 introns and 26 exons [18, 52]. This gene is one of the most commonly researched candidate genes in relation to EH resulting from I/D polymorphism at intron 16 [52].

##### 1.1.2.1. ACE Gene Polymorphism and EH

Several studies have observed I/D polymorphism in gene coding for ACE and its impact on susceptibility to EH in certain populations while not in others. A case–control study conducted among 428 South Indian individuals revealed that the populations carrying the D allele showed higher susceptibility to EH [53]. Another study conducted among 2040 individuals from Chinese Han populations suggests a similar relationship [54]. These findings also align with a meta-analysis of 57 studies, which comprises 32,862 participants of Asian, Caucasian and mixed descent. The study showcases that the D-allele of the ACE gene showed higher susceptibility to EH, while the Asian population among all showed a stronger association. The study also points out that the association was more pronounced in males across all populations [55].

Some of the older research done before 2010 also highlights a negative or no correlation between I/D polymorphism and EH (Table 2). However, this research, due to the smaller sample size, limits the generalisability and statistical power of the findings.

Interestingly, there is an exception for the population of African descent. Research in these groups has consistently shown a stronger correlation between polymorphism in ACE and EH. However, this hypothesis about the direct influence of genetic variability in the ACE gene on BP levels is not widely accepted for all populations but among those of African descent.

This notion is supported by a linkage and association study carried out on a large population of individuals with African ancestry that identified two specific SNPs (ACE8 and ACE4) in the ACE gene. These variations were found to be strongly linked with ACE concentration and had a notable impact on BP level [61].

#### 1.1.3. AT1R

Ang II is an important vasoconstrictor, consisting of two subtypes of the receptors (AT1 and AT2) on the cell surface. AT1 is the receptor through which most actions of Ang II are exerted [19, 62, 63]. The main receptor for Ang II in humans that mediates both vasoconstrictor action and growth-promoting effects is AT1R [64]. The AT1 gene, which codes for AT1R and has a length of 45.123 kb consisting of five exons and four introns, is found on the long arm of the chromosome 3 (3q21–q25) [3, 19, 61, 62]. A1166C transversion in the AT1R gene has been the topic of most research in relation to EH.

##### 1.1.3.1. AT1R Gene Polymorphism and EH

The AT1R gene exhibits five common polymorphisms (T573C, A1062G, A1166C, G1517T and A1878G) [65]. Among these, most studies have focused on A1166C gene polymorphism, which shows an A-to-C substitution at position 1166 in the AT1R gene, and these studies have revealed its association with EH [19].

**Figure 2:**
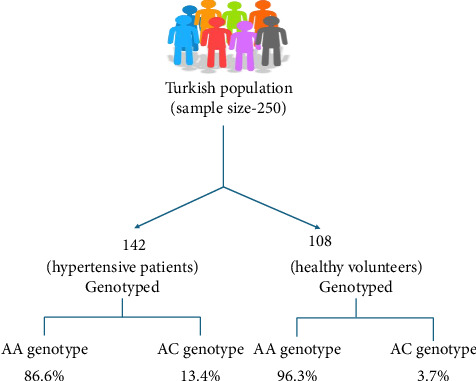
Illustration of AT1R (A1166C) gene polymorphism representing a higher prevalence of risk of EH in the patient carrier of the C genotype.

A case–control study conducted in the Gujarati Indian population with a sample size of 481 individuals carrying the C allele in AT1R showed high susceptibility to EH [66]. Another study among the South Indian population, comprising 200 cases and 200 controls, showed similar results [67]. A meta-analysis also indicated an increased possibility of EH by 1.18-fold and 1.15-fold in patients carrying the C allele and AC genotype. However, a decreased chance of EH was seen in patients carrying the A allele and AA genotype. Subgroup analysis also supported these findings in the Asian population [68]. Similarly, research conducted in the Turkish population with a sample size of 250 individuals carrying the C allele showed a positive relationship with EH (Figure 2) [19].

In contrast, there are a few studies carried out in diverse populations that show no association between A1166C gene polymorphism and EH. A study conducted in the population of Calabar and Uyo did not find a notable link between the A1166C gene and EH [69]. Similarly in Jordanian hypertensives, the A1166C polymorphism was not linked to the early onset of EH, but it did show an association in males (AC, CC) with strong family histories of EH and those with larger waist circumferences [62]. Similarly, some of the older research, as summarised in Table 3, highlights both positive and negative relationships of A1166C polymorphism and EH.

However, it is crucial to note that the majority of these studies show a correlation between A1166C gene polymorphism and EH, while some do not. Thus, it becomes important to explore this particular gene polymorphism with a larger sample size and a diverse population to precisely understand the relationship between genetic variation and EH.

#### 1.1.4. Aldosterone Synthase

The RAAS pathway is crucial for maintaining BP, and aldosterone is one of its main effectors [72]. In the production of aldosterone, aldosterone synthase is a crucial enzyme that is encoded by the CYP11B2 gene. It is a member of the cytochrome P450 family of enzymes, which spans over 7 kb of chromosome 8q24.3, each containing nine exons. It encodes for cytochrome P450, which is crucial for aldosterone synthesis, and Ang II, and potassium regulates its expression [73, 74].

Within CYP11B2, C344T is a common polymorphism where C-to-T substitution takes place [74]. Various studies on this polymorphism have demonstrated both positive and negative relationships with EH.

##### 1.1.4.1. Aldosterone Synthase (CYP11B2) C344T Polymorphisms and EH

CYP11B2 (C344T) has been associated with EH in several studies; a case–control study from Pakistan involving 400 individuals showed a significant association [75].

Studies conducted before 2015 in African-Americans, Latinos, Tibetans and other populations have also revealed associations with the C344T polymorphism and EH [20, 21, 76, 77]. The recent studies, however, show a negative relationship between C344T polymorphism and EH.

A study conducted involving 1776 participants among Tibetans (545), Dongxians (530) and Chinese Han (701) showed both positive and negative relationships. The positive association was observed in Tibetan women; however, no links were observed between C344T polymorphism and EH among the other two populations [21]. A meta-analysis of research conducted in the Chinese Han population also shows no association between this polymorphism and EH [78]. This reveals there is limited recent research supporting a positive relationship in diverse populations between C344T gene polymorphism and EH. Thus, a further exploration with a larger population size conducted in a diverse population is needed to clarify the interplay of C344T gene polymorphism and EH.

### 1.2. Pharmacogenetics

Pharmacogenetics is the study of how individual genetics affect drug responses, and this concept was first discussed 50 years ago. Since decades, drug development and patient treatment often consider large patient populations as homogeneous groups rather than considering the genetic variation among these patients.

According to numerous data points, 30%–60% of the patients fail the effective treatment of the disease [79] due to a lack of genetic considerations. This highlights a need for a more personalised approach to treatment. Pharmacogenetics thus promises the potential of personalising drug therapy, which shall lead to effective drug treatment depending on each patient's specific genetic profile and lower chances of having adverse drug reactions [80].

#### 1.2.1. Variability in Antihypertensive Drug Response

The significant variability in BP responses to antihypertensive drugs highlights the challenges in achieving consistent treatment outcomes in diverse patients. For instance, a study focused on the treatment of patients with severe EH shows varied BP responses to antihypertensive drugs. On the one hand, under placebo treatment, the systolic BP exhibited a notable increase of 28 mmHg in a few patients to a substantial decrease of 76 mmHg in others.

On the other hand, patients receiving active treatment displayed a different range of results, including an increase in SBP to 12 mmHg in some and a remarkable decrease to 76 mmHg in others. However, the results for diastolic pressure varied, from an increase of 28 mmHg to a decrease of 44 mmHg in placebo treatment and in active treatment, from an increase of 12 mmHg in some to a decrease of 60 mmHg in the rest [81]. The study clearly depicts the significant variability in BP response to both placebo and active drug treatment among individuals.

Another study carried out among two different racial groups (black and white) with hypertension also found the variability in response when treated with the same drug (quinapril), but the variability was seen within the racial groups rather than between the groups [82].

Different observations collectively highlight the need to move beyond the current ‘trial-and-error' approach in selecting pharmacological treatments for hypertension. To enhance treatment efficacy and precision, it becomes imperative to identify patient characteristics that influence BP response to specific medication classes [83]. This pursuit aims to tailor hypertension management strategies more precisely to individual patients' profiles, ultimately improving clinical outcomes.

#### 1.2.2. Drug Response: Genetic Contributions

The first cardiovascular medication, in which clinical reactions varied based on race, was antihypertensive medication. Diuretics and beta-blockers have different effects on decreasing BP depending on the ethnic group, as was discovered in the early 1980s. The outcome of the pharmacological therapy is frequently uncertain; it might range from having positive therapeutic effects to having negative therapeutic effects due to genetic variability.

**Figure 3:**
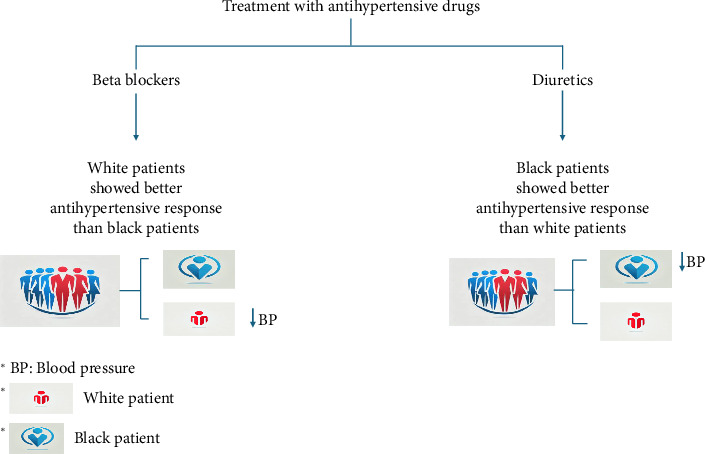
Illustration of antihypertensive drug variance among whites and blacks.

Ethnic variance in beta-blocker and ACE inhibitor responses imply that distinct mechanisms contribute to the development of EH in different ethnic populations. The Veterans Affairs Cooperative Trial provided the most compelling evidence of the discrepancy between white and black patients' responses to various antihypertensive drugs. Beta-blockers showed a slightly better antihypertensive effect on white patients than on black patients, whereas diuretics had a marginally better effect on black patients (Figure 3) [84].

#### 1.2.3. RAAS Gene Polymorphism and Antihypertensive Drug Response

##### 1.2.3.1. Renin Gene Polymorphism and Antihypertensive Drug Response

A study involving 461 whites and 297 blacks investigated the genetic factors influencing baseline plasma renin activity and found a specific single nucleotide polymorphism (rs3784921) in the SNN-TXNDC11 gene region. The G allele of rs3784921 was linked with higher baseline plasma renin activity in the whites and a reduced response in BP to hydrochlorothiazide [17].

Another study explored the response of the drug valsartan to RAAS polymorphism. Among five genetic variants examined, one, REN C-5312T, was associated with different responses to the drug. Patients carrying the CC genotype experienced a greater decrease in DBP, while the response rate to the medication was higher in the CT/TT genotype [85]. Likewise, another study that investigated the same genetic variant (REN C-5312T) and its response to valsartan and telmisartan also found that CT/TT carriers showed a lesser reduction in DBP as compared to the CC genotype [86].

These studies highlight the reduced efficacy of antihypertensive drugs to renin gene polymorphism, suggesting a potential for personalised medication considering the genetic makeup of an individual. Additionally, there is a need for more pharmacogenetic studies delving into the renin gene polymorphism and the drug response.

##### 1.2.3.2. ACE Gene Polymorphism and ACEI Response

ACE inhibitors function by blocking the conversion of Ang I to Ang II in tissues and blood vessels, resulting in vasodilation [32]. The ACE gene consists of 26 exons, located on chromosome 17's long arm [18]. Among several polymorphisms in the ACE gene, the effects of the ACE I/D polymorphism on pharmacogenetics have been the subject of numerous studies [32].

**Figure 4:**
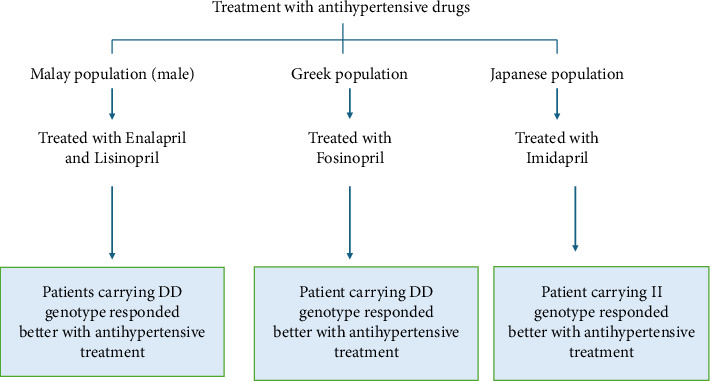
Illustration of variation in drug response among different populations.

A study was conducted on Malay male individuals (72 healthy individuals and 72 newly diagnosed hypertension individuals) to assess the response to ACE inhibitors (enalapril and lisinopril). It was found that patients with the DD genotype experienced a greater decrease in BP compared to those with ID and II genotypes, suggesting that the D allele may be a potential genetic marker for EH in Malay male subjects [28]. Another study conducted in a Greek population treated with fosinopril showed a similar response, with a significant decrease in BP among patients carrying the DD genotype compared to those with the ID and II genotypes [76]. However, a study conducted in the Japanese population showed a better antihypertensive response with imidapril in patients carrying the II genotype rather than the DD genotype (Figure 4) [87].

The studies above explored the relationship between I/D genotype and ACEI, in which DD genotype responded better to ACEI as compared to ID and II genotypes. However, a study conducted in the Japanese population showed contrasting results, hinting at a need to explore the genetic variation in different ethnicities and the drug response. Additionally, there is a need for larger sample sizes and greater emphasis on the generalisability of these findings to diverse populations to enhance the applicability of the results.

##### 1.2.3.3. AT1R Gene Polymorphism and AT1R Blocker Response

The human AT1R gene has been found to be highly polymorphic. Several studies have been conducted to understand the drug response with respect to A1166C gene polymorphism. In a retrospective study involving 281 hypertensive patients on valsartan treatment, a higher frequency of the C allele and AC/CC genotypes of the AT1R A1166C polymorphism was detected in patients with well-controlled BP compared to those with poorly controlled BP [33].

In another study involving Caucasians, a single dose of 25 mg of losartan was given to 66 voluntary individuals to determine the drug response to A1166C gene polymorphism. As a result, a decrease in the level of aldosterone was found in patients with the C allele compared to those with the AA genotype. Additionally, patients with the C allele showed better potency in lowering their BP than those with the AA genotype, wherein lowering aldosterone levels determines lowering BP [31].

Contrary to the above results, few studies have also highlighted no relationship between A1166C polymorphism and drug responses. For instance, a study involving 206 Caucasian hypertensive patients found that A1166C gene polymorphism did not influence the antihypertensive efficacy of telmisartan [34]. The findings emerging from SILVHIA trials also showed similar results where there was no significant association between aforementioned polymorphism and drug response [35].

These studies highlight that the A1166C gene polymorphism is linked to the increase/decrease in the efficacy of AT1R blockers, while other studies show no association. Thus, this poses a need for more population-based research with an adequate sample size to develop a personalised approach to optimise treatment.

##### 1.2.3.4. RAAS Gene Polymorphism and Response to Other Antihypertensive Drugs

Research on genetic determinants of BP response to antihypertensive treatments has yielded intriguing insights. A study aimed to explore association between the RAAS gene polymorphism and their ability to predict BP-lowering responses to atenolol and irbesartan. Both treatments showed similar mean BP reductions. The irbesartan-treated group exhibited a notable effect in individuals with a specific genotype (ACE gene I allele), resulting in a significant reduction in DBP compared to those with the D allele. This genotype-specific response was not observed in the atenolol group [35].

However, some contradictory results exist, as exemplified by a large population-based cohort study in the Netherlands. This study investigated the impact of I/D polymorphism in the ACE gene on BP responses to various antihypertensive drugs, including diuretics, beta-blockers, calcium channel blockers, and ACE inhibitors, and found no significant modification of mean BP differences associated with this genetic variation [88].

Additionally, research conducted in Chinese patients as part of a benazepril post-market surveillance trial examined genetic variations in the AGT, AT1R and AT2R genes. The study identified associations between the AGT single nucleotide polymorphism rs7079 and AT1R haplotype with BP reduction in response to ACE inhibitors [64]. Another study conducted in the North Indian populace showcased that the individual with the TT genotype exhibited a high BP lowering response to an ACE inhibitor (Enalapril) as compared to those with the MT and MM genotypes [89].

Some studies have also explored genetic variation with different populations in BP response to specific antihypertensive medications. A study involving hypertensive African Americans and non-Hispanic whites showed that the effects of A1166C polymorphism of the AT1R gene could influence the BP response to thiazide diuretics [90].

Another study in non-Hispanic men and women with EH who underwent hydrochlorothiazide monotherapy revealed distinct impacts of ACE I/D polymorphism in response to hydrochlorothiazide [91]. A study conducted in the Chinese Han ethnic population also uncovered a similar association to hydrochlorothiazide treatment [92]. These studies thus showcase a complexity of genetic influence on BP response to antihypertensive drugs.

## 2. Conclusion

The review highlights the role of genetic polymorphism and its association with EH in RAAS. The RAAS gene polymorphisms in AGT, ACE, AT1R and aldosterone synthase have shown increased susceptibility to EH. These polymorphisms have also influenced the therapeutic impact of antihypertensive drugs like hydrochlorothiazide, telmisartan, atenolol, etc. The majority of studies presented in this review highlight a need for research conducted in diverse populations with a larger sample size to understand the interplay of genetic variation, EH and antihypertensive drugs to pave a path for a more personalised approach towards drug therapy in managing EH.

### 2.1. Summary and Future Perspective

Genetic polymorphisms account for interindividual variability and abnormal responses to antihypertensive drugs. The correlation between genetic changes in the primary RAAS components and EH has been the subject of numerous investigations. Therefore, RAAS suppression is the therapeutic strategy for BP maintenance, although it has been found that due to genetic variability, not all patients achieve BP management adequately.

Differences in ethnicities and their response to ACE inhibitors and AT1R blockers show involvement of several pathways causing EH in several ethnic groups. Many drugs have been demonstrated to be effective in treating EH; however, each patient will respond to these drugs differently. There are currently no efficient ways to tailor antihypertensive medication. Thus, the identification of causative genes and genetic variants will greatly benefit understanding the genotype–phenotype link. This could lead to earlier intervention and better patient care.

Our exploration to understand association between RAAS gene polymorphism and EH showcased limited availability of recent research exploring renin gene polymorphism and its impact on EH. This review has also looked at all types of research findings, both positive and no association of RAAS polymorphism with EH, where recent research with a larger sample size showed a higher positive relationship as compared to others. However, in the case of aldosterone synthase C344T polymorphism, the majority of research conducted shows no associations between this variation and EH.

To reach a more accurate conclusion, more research concentrating on gene polymorphism and its relationship with therapeutic effectiveness is needed. However, in this article, the significance of some important RAAS gene polymorphisms, their interrelation to EH and their impact on antihypertensive drug therapies is summarised.

## Figures and Tables

**Table 1:** The AGT gene M235T polymorphism and its association with EH.

Gene	Polymorphism	Study population	Sample size	Main findings	Reference
AGT	M235T	Romanian	Cases = 38 Controls = 21	M235T variant more frequently present in hypertensive patients (81.7%) compared to control group (66.66%)	[43]
AGT	M235T	Indian	Cases = 279 Controls = 200	Certain genotypes and alleles of the M235T variants were more prevalent in female hypertensive patients, suggesting a potential gender-specific effect on EH risk	[44]
AGT	M235T	Dravidian	Cases: 211 Controls: 211	M235T variant is significantly associated with EH, particularly in female patients	[15]
AGT	M235T	Taiwanese	Cases: 102 Control: 49	M235T variant is linked with EH in Taiwanese population	[45]
AGT	M235T	Minnesotans	Cases: 104 Control: 195	M235T variant may not have a significant role in the development of EH in this group	[46]
AGT	M235T	Lebanese	Cases: 270 Control: 124	The TT genotype was found to be less prevalent in cases compared to the control group	[47]

*Note:* The table summarises some of the research findings of M235T gene polymorphism and EH. These studies yield the study conducted before 2015, which showcases both positive and negative relationships of polymorphism and EH. These studies, however, have a smaller sample size, thus limiting their generalisability.

**Table 2:** ACE gene I/D polymorphism and EH.

Gene	Polymorphism	Study population	Sample size	Main findings	Year	Reference
ACE	I/D	Korean population	Cases: 426 Control: 461	I/D polymorphism does not show a clear association with EH in the population	2004	[56]
ACE	I/D	Tibetan population	Case: 106 Control: 135	The study's findings suggest a significant link between the D allele and EH in Tibetan women and not in Tibetan men	2002	[57]
ACE	I/D	Cuban population	Cases: 243 Control: 407	ACE I/D polymorphism is not linked with EH in the population	2007	[58]
ACE	I/D	Punjabi population of Pakistan	344 hypertensive and normotensive patients	ACE gene I/D polymorphism is not linked with EH in Punjabi population	2009	[59]
ACE	I/D	Bangladesh population	Cases: 44 Control: 59	The DD genotype was linked with the highest value of mean SBS and mean DBP in men but not in women	2002	[60]

*Note:* The table summarises the findings of studies conducted in diverse populations where ACE gene I/D polymorphism does not show a significant relationship either with some population or one gender within the population studied.

**Table 3:** AT1R gene A1166C polymorphism and EH.

Gene	Polymorphism	Study population	Sample size	Main findings	Reference
AT1R	A1166C	Japanese population	1207	A1166C gene polymorphism is not a major genetic predisposing factor for EH in the population	[70]
AT1R	A1166C	Han, Tibetan and Yi populations of China	Cases: 446 Control: 302	A1166C gene polymorphism may play a role in the development of EH in Tibetan males but not in the Han or Yi population	[71]
AT1R	A1166C	Indian (New Delhi)	Cases: 250 Control: 250	The C allele of the A1166C polymorphism in the AT1R gene is associated with EH	[8]
AT1R	A1166C	Serbian population	Cases: 100 Control: 198	A1166C polymorphism of the AT1R gene may play a role in EH among males in the population	[72]

*Note:* The table summarises the findings of studies conducted in Japanese, Chinese, Tibetans, Indians and Serbian populations, where the Japanese population shows no association between A1166C gene polymorphism and EH, while other populations show a positive relationship.

## Data Availability

This review is based on previously published articles, which have been duly cited in the manuscript. No new data were generated for this study.

## Nomenclature

ACE: Angiotensin converting enzyme
AGT: Angiotensinogen
AT1R: Angiotensin II type 1 receptor
Ang I: Angiotensin I
Ang II: Angiotensin II
BP: Blood pressure
EH: Essential hypertension
I/D: Insertion/deletion
RAAS: Renin–angiotensin–aldosterone system
